# 基于DIA蛋白组学构建脓毒症性凝血病诊断模型的临床研究

**DOI:** 10.3760/cma.j.cn121090-20241219-00579

**Published:** 2025-01

**Authors:** 奇 陈, 景春 宋, 小雷 万, 俊杰 曾, 晓敏 宋, 林翠 钟, 龙平 何

**Affiliations:** 解放军联勤保障部队第九〇八医院重症医学科/南昌大学附属长城医院重症医学科，南昌 330002 Intensive Care Unit, the 908th Hospital of Chinese PLA Logistical Support Force, Changcheng Hospital Affliated to Nanchang University, Nanchang 330002, China

**Keywords:** 脓毒症性凝血病, 蛋白组学, 诊断模型, 列线图, 血管生成素, Sepsis-induced Coagulopathy, Proteomics, Diagnostic Model, Nomogram, Angiogenin

## Abstract

**目的:**

采用数据非依赖型采集（DIA）蛋白质组学技术分析脓毒症性凝血病（SIC）的血浆蛋白表达，筛选典型生物标志物并构建诊断模型。

**方法:**

前瞻性观察研究纳入重症医学科46例成年脓毒症患者，收集临床资料并按照SIC标准分为普通脓毒症组（26例）和SIC组（20例），采集血浆标本进行蛋白组学检测并进行生信物信息学分析，应用LASSO、随机森林筛选差异表达蛋白（DEP），根据筛选结果构建SIC诊断模型，并进行受试者工作特征（ROC）曲线分析。

**结果:**

基线资料显示，SIC患者的凝血酶原时间较脓毒症组明显延长，血小板计数明显降低，D-二聚体、纤维蛋白降解产物、血乳酸、SOFA评分和APACHE Ⅱ评分较脓毒症组患者明显升高（*P*<0.05）。DIA蛋白组学共鉴定出2 637个蛋白，以表达倍数>1.5倍且*P*<0.05为筛选标准，共筛选出240个DEP，包括81个上调DEP和159个下调DEP。亚细胞定位分析表明，DEP主要在细胞外和细胞核；GO注释显示，DEP在生物过程方面主要参与细胞生理、生物调节和应激反应过程；分子功能方面主要参与生物分子互作和催化作用；结构域注释显示DEP以免疫球蛋白V区为主，是特异识别和结合抗原的部分；KEGG富集分析显示，DEP主要富集在自然杀伤细胞介导的细胞毒性反应、糖基磷脂酰肌醇锚定蛋白合成通路、肿瘤坏死因子信号通路和NF-κB信号通路。对DEP进行LASSO回归和随机森林，筛选出血管生成素、凝集素家族成员10两个变量，且以此构建的SIC诊断模型的ROC曲线下面积为0.896，特异度为0.731，灵敏度为0.900。

**结论:**

由血管生成素、凝集素家族成员10构建的诊断模型可准确诊断SIC。

## Abstract (en)

**Objective:**

This study used data-independent acquisition（DIA）proteomics to analyze plasma protein expression in sepsis-induced coagulopathy（SIC）, identify key biomarkers, and develop a diagnostic model.

**Methods:**

This prospective study included 46 adult sepsis patients from the intensive care unit. Patients were categorized into a general sepsis group（*n*＝26）and an SIC group（*n*＝20）based on established SIC criteria. Plasma samples underwent proteomic and bioinformatics analyses to identify differentially expressed protein（DEP）using LASSO regression and Random Forest. A diagnostic model was constructed and assessed via receiver operating characteristic (ROC) curve analysis.

**Results:**

The baseline data revealed that SIC patients exhibited longer prothrombin times, lower platelet counts, and higher D-dimer, fibrin degradation products, blood lactate, SOFA scores, and APACHE Ⅱ scores compared with general sepsis patients（*P*<0.05）. DIA proteomics identified 2 637 proteins, with 240 DEP meeting the criteria（fold change >1.5, *P*<0.05）, including 81 upregulated and 159 downregulated DEP. Subcellular localization analysis revealed that DEPs were predominantly extracellular and nuclear. Gene ontology（GO）annotation showed that DEP were mainly involved in cellular physiology, biological regulation, and stress response processes in biological processes. Domain annotation revealed a predominance of immunoglobulin V regions in DEP, which are crucial for antigen recognition and binding. KEGG enrichment analysis showed significant enrichment of DEP in pathways related to natural killer cell-mediated cytotoxicity, glycosylphosphatidylinositol anchor biosynthesis, tumor necrosis factor signaling, and NF-κB signaling. LASSO regression identified angiogenin and C-type lectin domain family 10 member A as key DEP. The SIC diagnostic nomogram showed an area under the curve of 0.896, with 0.731 specificity and 0.900 sensitivity.

**Conclusion:**

The nomogram incorporating angiogenin and C-type lectin domain family 10 member A provides an accurate tool for SIC diagnosis.

脓毒症是指宿主对感染的异常反应导致的致死性器官功能障碍[1]。美国每年约170万成人发生脓毒症，约60余万在院内死亡[2]。中国重症医学质控中心报道，在重症医学科收治的成人脓毒症性休克患者的在院死亡率超过50％[3]。脓毒症时感染和炎症因素极易导致血管内皮损伤，从而发生以血小板减少、凝血紊乱和纤溶异常为特征的脓毒症性凝血病（Sepsis-induced coagulopathy, SIC）[4]。据统计，我国67.9％的脓毒症患者可合并SIC，SIC易进展为弥散性血管内凝血（DIC），导致SIC患者死亡率显著升高[5]–[6]。为了实现SIC患者的早期诊断，国际血栓与止血学会（International Society on Thrombosis and Haemostasis，ISTH）专门发布了由序贯器官衰竭评分（SOFA评分）、INR和血小板计数三个指标组成的SIC诊断积分法[7]。SIC诊断标准的最大优点是纳入指标非常普及，临床易于执行，但也导致SIC诊断标准的灵敏度和特异度不够理想[8]。因此，寻找更加灵敏、准确的诊断标志物一直是SIC的研究热点之一。

数据非依赖型采集（DIA）蛋白质组学能大规模检测蛋白质，具有高定量准确性和高数据重现性，已被广泛用于挖掘疾病机制、筛选早期诊断标志物和发现治疗靶点的相关研究，但在SIC方面尚未见报道[9]。因此，本研究拟采用DIA蛋白组学技术检测SIC患者血浆中蛋白表达的变化，探讨SIC的发病机制并筛选有意义的差异表达蛋白（Differentially expressed proteins，DEP），为早期精准诊断SIC提供依据。

## 病例与方法

一、研究对象和分组

本研究属于前瞻性观察性研究，共纳入2023年11月至2024年5月联勤保障部队第九〇八医院重症医学科收治的46例脓毒症患者。纳入标准：符合脓毒症3.0诊断标准[10]。排除标准为符合以下任意一条：①年龄<18岁；②转入ICU不足24 h或转出患者；③血液系统肿瘤患者；④既往肝病病史；⑤既往遗传性凝血异常。根据是否满足中国SIC诊断标准[11]，将46例脓毒症患者分为普通脓毒症组（26例）和SIC患者（20例）。本研究经联勤保障部队第九〇八医院伦理委员会批准（伦理批号：908yyLL028），并征得患者家属知情同意。

二、收集基础资料

收集患者的基础资料包括年龄、性别，入科2 h内的血常规（包括白细胞计数、中性粒细胞计数、淋巴细胞计数、红细胞计数和血小板计数），血生化（包括肝肾功能指标，具体为丙氨酸转氨酶、天冬氨酸转氨酶、总胆红素和肌酐），C-反应蛋白，乳酸，常规凝血指标（包括凝血酶原时间、活化部分凝血活酶时间、凝血酶时间、纤维蛋白原、纤维蛋白原降解产物和D-二聚体），并计算入科时的急性生理与慢性健康（Acute physiology and chronic health evaluation Ⅱ，APACHE Ⅱ）评分和SOFA评分。

三、蛋白提取与酶解

血浆复融离心后，加入适量裂解液提取蛋白，采用BCA法进行蛋白定量。各样品分别取20 µg蛋白质加入适量缓冲液，沸水浴5 min，进行SDS-PAGE电泳，考马斯亮蓝R-250染色。所有样品取适量蛋白，混合成Pool样品，用于构建spectral library，并采用过滤辅助样品制备法酶解。酶解后样品肽段进行高pH反相肽分离。肽段冻干后加入40 µl 0.1％甲酸溶液复溶，通过280 nm处的吸光度（*A*）值测定样品的肽段浓度。Pool样品肽段和酶解肽段中分别加入适量iRT标准肽段，进行DIA质谱检测。

四、质谱分析

采用纳升流速Evosep one系统（丹麦Evosep公司产品）进行色谱分离，纳米级高效液相色谱分离后的样品用timsTOF质谱仪（德国布鲁克公司产品）进行DIA质谱分析。DIA数据采用Spectronaut软件进行数据处理。搜库（UniProt）完成后，对结果进行样本质控评价、定量分析和DEP筛选。DEP筛选标准：以差异倍数（Fold change，FC）>1.5、*P*<0.05作为显著上调的变化阈值，FC<−1.5、*P*<0.05为显著下调的变化阈值。

五、生物信息学分析

对DEP进行亚细胞结构注释、结构域注释、GO注释、KEGG通路富集分析。

六、统计学处理

所有临床数据采用SPSS 27.0统计软件进行分析。计数资料以构成比或频数表示，组间比较采用*χ*^2^检验。计量资料以单样本S-W法进行正态分布检验，符合正态分布的数据以均数±标准差（*x*±*s*）表示；非正态分布的数据以中位数（四分位数）［*M*（*Q*_1_，*Q*_3_）］表示。满足正态分布且方差齐者组间比较采用*t*检验；不满足者组间比较采用Mann-Whitney *U*检验。以*P*<0.05为差异有统计学意义。使用R包“random Forest”进行随机森林。对DEP使用CNSknowall平台进行LASSO回归、受试者工作特征（Receiver operating characteristic, ROC）曲线分析、列线图和决策曲线分析的绘制。

## 结果

一、基线资料

如表1所示，炎症指标中，SIC患者的白细胞计数和中性粒细胞计数较脓毒症组上升。器官功能方面，SIC患者的肌酐水平增高，提示肾功能受损。疾病危重程度的评估上，SIC患者的血乳酸浓度、SOFA评分和APACHE Ⅱ评分均高于脓毒症组，表明病情更加严重。凝血功能方面，SIC患者的凝血酶原时间延长，血小板计数、纤维蛋白原水平显著降低，而D-二聚体、纤维蛋白降解产物水平升高，提示存在明显的凝血功能障碍。

**表1:** 脓毒症与脓毒症性凝血病（SIC）患者的基线资料

指标	脓毒症组（26例）	SIC组（20例）	统计量	*P*值
年龄［岁，*M*（*Q*_1_, *Q*_3_）］	78.5（59.5, 87）	83（71, 84.5）	−0.189	0.850
男性［例（％）］	17（65.38）	10（50.00）	1.104	0.293
WBC［×10^9^/L，*M*（*Q*_1_, *Q*_3_）］	7.20（5.78, 10.15）	15.00（9.63, 15.80）	−3.503	<0.001
ALC（×10^9^/L，*x*±*s*）	0.88±0.39	0.91±0.55	−0.227	0.822
ANC［×10^9^/L，*M*（*Q*_1_, *Q*_3_）］	5.75（4.78, 8.35）	12.85（7.85, 14.95）	−3.458	<0.001
RBC（×10^12^/L，*x*±*s*）	3.55±0.88	3.28±0.97	0.971	0.338
PLT（×10^9^/L，*x*±*s*）	205.30±47.40	98.15±74.92	5.593	<0.001
PT［s，*M*（*Q*_1_, *Q*_3_）］	13.1（12.2, 14.2）	17.6（16.4, 18.7）	−5.420	<0.001
APTT［s，*M*（*Q*_1_, *Q*_3_）］	33.4（27.6, 38.5）	35.7（30.3, 46.9）	−1.571	0.080
TT［s，*M*（*Q*_1_, *Q*_3_）］	16.2（14.8, 16.8）	17.3（14.9, 19.6）	−1.497	0.134
FIB（g/L，*x*±*s*）	3.99±0.90	2.82±0.92	4.331	<0.001
纤维蛋白降解产物［µg/ml，*M*（*Q*_1_, *Q*_3_）］	3.39（2.46, 4.08）	6.63（4.09, 9.18）	−4.321	<0.001
D-二聚体［µg/ml，*M*（*Q*_1_, *Q*_3_）］	1.04（0.825, 1.68）	2.63（1.45, 4.10）	−4.144	<0.001
肌酐［µg/ml，*M*（*Q*_1_, *Q*_3_）］	70.35（45.05, 95.30）	101.40（74.15, 159.73）	−2.726	0.006
丙氨酸转氨酶［U/L，*M*（*Q*_1_, *Q*_3_）］	41.50（26.23, 64.35）	48.01（14.70, 62.78）	−0.244	0.807
天冬氨酸转氨酶［U/L，*M*（*Q*_1_, *Q*_3_）］	38.3（25.6, 55.5）	47.4（29.2, 67.1）	−0.909	0.364
C反应蛋白［mg/L，*M*（*Q*_1_, *Q*_3_）］	53.8（36.9, 114.7）	79.2（44.5, 181.0）	−0.997	0.319
乳酸［mmol/L，*M*（*Q*_1_, *Q*_3_）］	1.5（1.3, 1.9）	2.3（1.4, 3.1）	−2.359	0.017
SOFA评分［*M*（*Q*_1_, *Q*_3_）］	5.00（3.75, 7.00）	9.00（6.00, 11.00）	−4.037	<0.001
APACHE Ⅱ评分（*x*±*s*）	18.69±4.60	22.35±4.67	−2.655	0.011

**注** ALC：淋巴细胞绝对计数；ANC：中性粒细胞绝对计数；PT：凝血酶原时间；APTT：活化部分凝血活酶时间；TT：凝血酶时间；FIB：纤维蛋白原；SOFA：序贯器官衰竭评分；APACHE Ⅱ：急性生理与慢性健康Ⅱ评分

二、差异表达蛋白的筛选

质谱检测结果以韦恩图显示共鉴定到2 637个重叠蛋白（图1A），本项目所有样本及质量控制（QC）样本内蛋白的定量强度分布以抖动图形式展示如下（图1B）。以FC>1.5、*P*<0.05为上调蛋白变化阈值，FC<−1.5、*P*<0.05为下调蛋白变化阈值，共筛选出240个DEP，其中上调蛋白81个，下调蛋白159个。以FC值和*P*值两个因素为标准绘制火山图（图1C），以热图形式采用层次聚类算法对DEP进行聚类分析（图1D）。

**图1:**
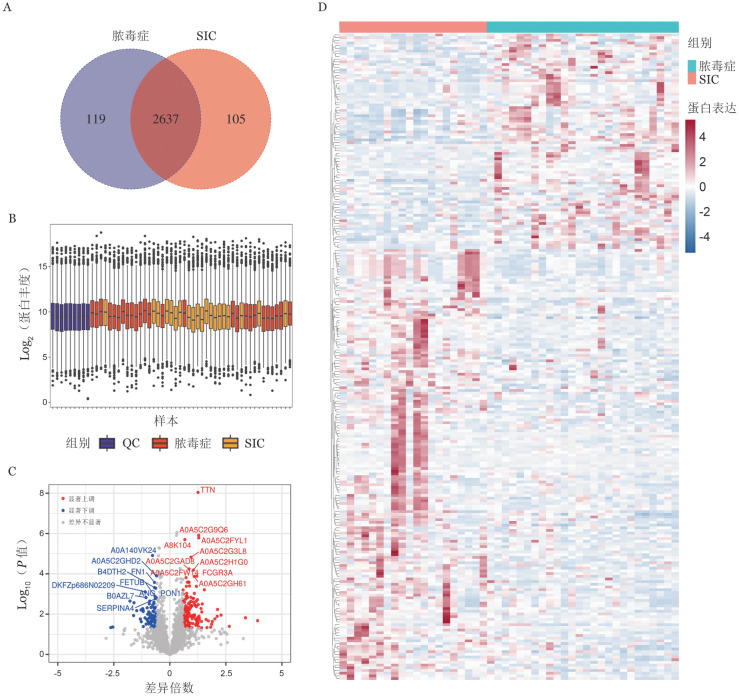
蛋白的鉴定和质控及差异蛋白筛选与聚类分析 A 韦恩图显示脓毒症与SIC重叠蛋白；B 样本强度定量抖动分布图；C 火山图（红色标注上调蛋白，蓝色标注下调蛋白，标示出差异最显著的前10个蛋白）；D 聚类分析图（240个差异蛋白在脓毒症与SIC中分别聚为两个独立的簇，表明差异蛋白在两组之间存在显著的表达模式差异） **注** SIC：脓毒症性凝血病样本；QC：质控样本

三、生物信息学分析

对所有DEP进行亚细胞定位分析，结果显示DEP主要在细胞外和细胞核（图2A）。结构域注释结果显示，差异蛋白结构域绝大多数聚集在免疫蛋白的V区（图2B）。GO功能注释显示，在生物过程方面DEP主要参与细胞生理、生物调节、应激反应过程、免疫反应过程，在分子功能方面DEP主要参与生物分子互作和催化作用；在细胞组成方面主要分布在细胞外区域（图2C）。KEGG通路富集分析结果显示，DEP主要富集在自然杀伤细胞介导的细胞毒性、糖基磷脂酰肌醇锚定蛋白生物合成通路、肿瘤坏死信号通路和NF-κB信号通路（图2D）。

**图2:**
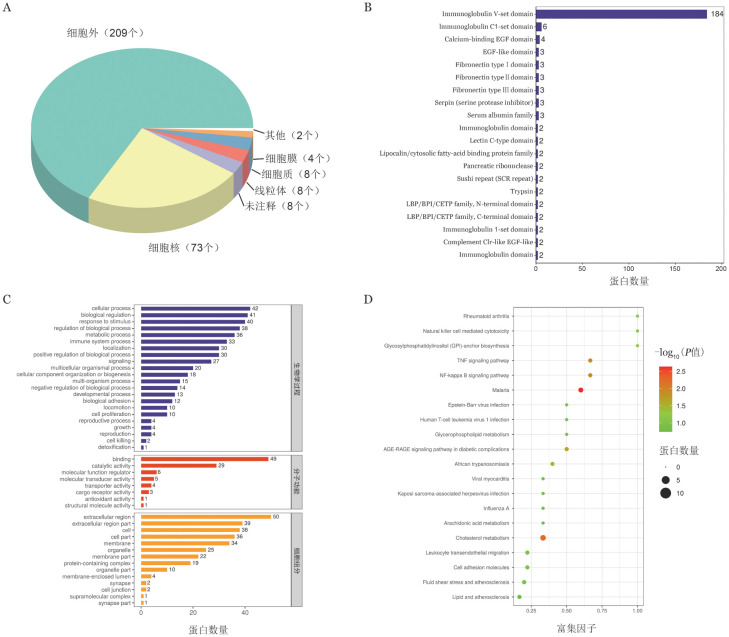
240个差异蛋白的生物信息学分析结果 A 亚细胞定位分析；B 结构域注释；C GO功能注释；D KEGG富集

四、生物标志物筛选

为寻找有临床价值的诊断标志物，首先对240个DEP进行初筛。排除无基因名称的免疫球蛋白片段后，采用LASSO回归进行筛选，当Log（λ）为−3.23时共筛选出11个DEP（图3A），随机森林筛选出18个DEP（图3B），两种方法重叠出6个DEP（图3C）。对这6个蛋白进行相关性分析（图3D），将相关系数绝对值大于0.4的蛋白剔除1个以避免出现功能相似标志物，筛选出P03950（血管生成素）和A0A024R9J3（凝集素家族成员10）。

**图3:**
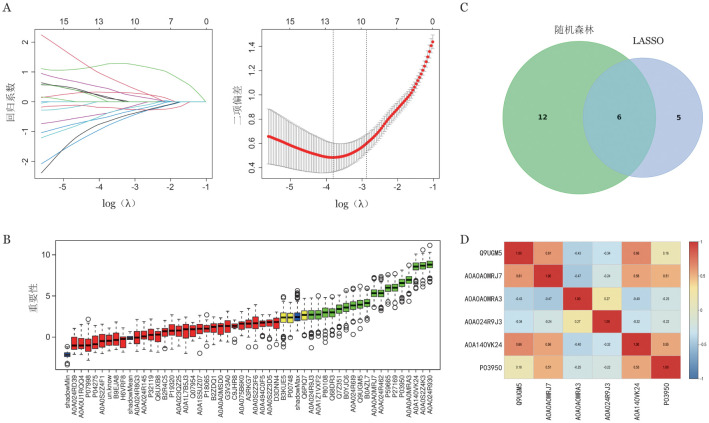
差异蛋白（DEP）的变量筛选过程 A 应用LASSO回归筛选变量（通过λ对变量进行筛选，最终纳入11个DEP）；B 应用随机森林模型筛选变量（绿色表示重要变量，红色表示不重要变量，黄色为暂定变量，蓝色代表阴影属性的最小、平均和最大Z值，最终筛选出18个重要DEP）；C 韦恩图筛选LASSO和随机森林中共有的重要标志物；D 相关性分析（将相关系数绝对值大于0.4的蛋白剔除1个以避免出现功能相似标志物）

五、诊断模型的构建

针对血管生成素和凝集素家族成员10蛋白构建SIC诊断模型并绘制列线图（图4A）。校准曲线显示平均绝对误差0.04（图4B）。临床决策曲线分析显示该预测模型的临床效益良好（图4C）。该诊断模型的ROC曲线下面积为（AUC）0.896，特异度为0.731，灵敏度为0.900（图4D）。

**图4:**
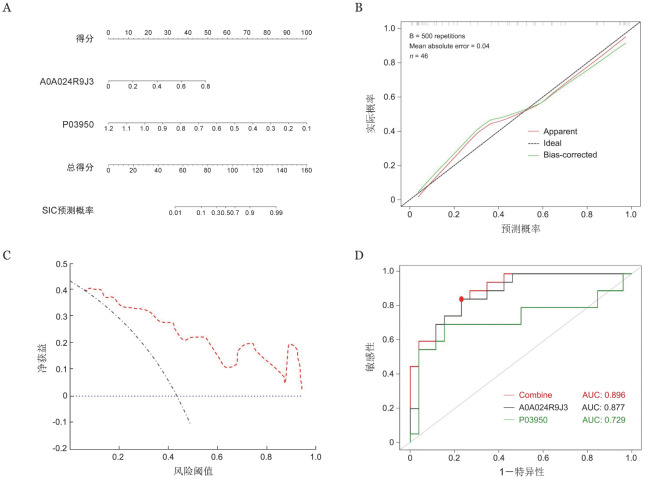
脓毒症性凝血病（SIC）诊断模型的效能评价 A SIC诊断列线图；B 校准曲线；C 临床决策曲线；D 受试者工作特征（ROC）曲线

## 讨论

本研究在国内外率先应用DIA蛋白组学筛选SIC患者早期诊断标志物。经生物信息学分析证实，SIC患者的240个DEP主要参与凝血异常、免疫反应和炎症通路，由LASSO回归和随机森林分析发现主要DEP为血管生成素和凝集素家族成员10。由其构建的SIC诊断模型的AUC为0.896，灵敏度为0.900，特异度为0.731。2024年，Shen等[12]报道针对22例脓毒症患者和10名健康人进行蛋白组学研究，经鉴定发现174个DEP，其功能主要集中于炎症反应、细胞外基质的代谢、细胞分泌物的分泌、细胞的活化和免疫反应。应用该研究筛选出的骨桥蛋白1诊断脓毒症的AUC为0.878。2024年12月，Palmowski等[13]报道224例存活和139例死亡的脓毒症患者，经鉴定发现的87种DEP功能主要集中于血液凝固、免疫反应和补体激活有关。其使用随机森林模型预测脓毒症30 d生存率的AUC为0.75。与同类研究相比，本研究针对脓毒症是否合并凝血病分组，与死亡脓毒症、普通脓毒症和健康人的相关数据分析可知，随着脓毒症的病情加重，凝血异常和免疫紊乱明显加重。本研究得出的SIC诊断模型效能也明显优于同类研究。

血管生成素是核糖核酸酶超家族成员，由123个氨基酸组成，循环浓度约400 ng/ml，因其能促进血管生成而得名，目前发现血管生成素具有较强的抗炎活性[14]。血管生成素能抑制成纤维细胞生成TNF-α，进而降低NF-κB、IL-6、IL-8和TNF-α受体等表达水平，从而发挥抗炎作用[15]。作为原始的核糖核酸酶，血管生成素还具有促进凝血因子活化的作用[16]。此外，血管生成素能够在微摩尔浓度下对白色念珠菌、肺炎链球菌和结核分枝杆菌产生杀菌效应。凝集素家族成员10是整合素家族成员，主要在肝脏、肾上腺、胸腺、脊髓和肾脏中表达，可在免疫反应和炎症过程中发挥关键作用[17]。凝集素家族成员10是一种模式识别受体，能够通过病原体相关分子模式（Pathogen-associated molecular patterns，PAMP）和损伤相关分子模式（Damage-associated molecular patterns，DAMP），促进免疫细胞的激活，从而增强宿主对感染的反应。与健康对照者相比，脓毒症患者的血浆Collectin-10可显著升高[18]。在新冠病毒感染患者中，凝集素家族成员10被报道能促进组织因子的表达和凝血酶原的活化，调节血小板功能[19]。此外，肝衰竭患者血浆Collectin-10水平也会显著升高，且升高者发生门静脉血栓的风险也更高[20]。本研究筛选出的主要DEP血管生成素和凝集素家族成员10主要集中在凝血异常和免疫紊乱的病理生理过程，这也说明SIC的主要病理生理机制是炎症与凝血障碍的交互作用。

本研究的不足之处主要有以下几点：第一，所筛选标志物均基于质谱方法测定，还需经过酶联免疫吸附法等多种实验方法验证结果及参考值范围。第二，本模型主要基于单中心研究数据，还未经过外部验证。第三，本研究的样本量相对偏小。

综上所述，本研究基于DIA蛋白组学筛选典型DEP，构建使用凝集素家族成员10和血管生成素组建的SIC诊断模型是全新高效的SIC诊断工具。

## References

[1] Evans L, Rhodes A, Alhazzani W (2021). Surviving sepsis campaign: international guidelines for management of sepsis and septic shock 2021[J]. Intensive Care Med.

[2] Fay K, Sapiano M, Gokhale R (2020). Assessment of Health Care Exposures and Outcomes in Adult Patients With Sepsis and Septic Shock[J]. JAMA Netw Open.

[3] Xie J, Wang H, Kang Y (2020). The Epidemiology of Sepsis in Chinese ICUs: A National Cross-Sectional Survey[J]. Crit Care Med.

[4] 宋 景春, 丁 仁彧, 吕 奔 (2024). 脓毒症性凝血病诊疗中国专家共识(2024版)[J]. 解放军医学杂志.

[5] Singh B, Hanson AC, Alhurani R (2013). Trends in the incidence and outcomes of disseminated intravascular coagulation in critically ill patients (2004-2010): a population-based study[J]. Chest.

[6] Ding R, Wang Z, Lin Y (2018). Comparison of a new criteria for sepsis-induced coagulopathy and International Society on Thrombosis and Haemostasis disseminated intravascular coagulation score in critically ill patients with sepsis 3.0: a retrospective study[J]. Blood Coagul Fibrinolysis.

[7] Iba T, Levy JH, Warkentin TE (2019). Diagnosis and management of sepsis-induced coagulopathy and disseminated intravascular coagulation[J]. J Thromb Haemost.

[8] Tanaka C, Tagami T, Kudo S (2021). Validation of sepsis-induced coagulopathy score in critically ill patients with septic shock: post hoc analysis of a nationwide multicenter observational study in Japan[J]. Int J Hematol.

[9] Heil LR, Fondrie WE, McGann CD (2022). Building Spectral Libraries from Narrow-Window Data-InDEPsendent Acquisition Mass Spectrometry Data[J]. J Proteome Res.

[10] Singer M, Deutschman CS, Seymour CW (2016). The Third International Consensus Definitions for Sepsis and Septic Shock (Sepsis-3)[J]. JAMA.

[11] 中国医药教育协会 (2024). T/CMEAS 019-2024. 凝血障碍诊断规范[S].

[12] Shen YZ, Xiong W, Hu YC (2024). SPP1 is a plasma biomarker associated with the dia gnosis and prediction of prognosis in sepsis[J]. Sci Rep.

[13] Palmowski L, Weber M, Bayer M (2025). Mortality-associated plasma proteome dynamics in a prospective multicentre sepsis cohort. EBioMedicine[J].

[14] Mao M, Chen W, Ye D (2024). Research progress on the structure, function, and use of angiogenin in malignant tumours[J]. Heliyon.

[15] Noschka R, Gerbl F, Löffler F (2020). Unbiased Identification of Angiogenin as an Endogenous Antimicrobial Protein With Activity Against Virulent Mycobacterium tuberculosis[J]. Front Microbiol.

[16] Garnett ER, Raines RT (2022). Emerging biological functions of ribonuclease 1 and angiogenin[J]. Crit Rev Biochem Mol Biol.

[17] Keshi H, Sakamoto T, Kawai T (2006). Identification and characterization of a novel human collectin CL-K1[J]. Microbiol Immunol.

[18] García-Laorden MI, Hernández-Brito E, Muñoz-Almagro C (2020). Should MASP-2 Deficiency Be Considered a Primary Immunodeficiency? Relevance of the Lectin Pathway[J]. J Clin Immunol.

[19] Bumiller-Bini V, de Freitas Oliveira-Toré C, Carvalho TM (2021). MASPs at the crossroad between the complement and the coagulation cascades - the case for COVID-19[J]. Genet Mol Biol.

[20] Zhang M, Jing Y, Xu W (2023). The C-type lectin COLEC10 is predominantly produced by hepatic stellate cells and involved in the pathogenesis of liver fibrosis[J]. Cell Death Dis.

